# PhenX RISING: real world implementation and sharing of PhenX measures

**DOI:** 10.1186/1755-8794-7-16

**Published:** 2014-03-20

**Authors:** Catherine A McCarty, Wayne Huggins, Allison E Aiello, Robert M Bilder, Ahmad Hariri, Terry L Jernigan, Erik Newman, Dharambir K Sanghera, Timothy J Strauman, Yi Zeng, Erin M Ramos, Heather A Junkins

**Affiliations:** 1 Essentia Institute of Rural Health, Maildrop: 6AV-2, 502 East Second Street, Duluth, MN 55805, USA; 2 RTI International, Research Triangle Park, Durham, NC USA; 3 University of North Carolina at Chapel Hill, Chapel Hill, North Carolina, USA; 4 University California Los Angeles, Los Angeles, CA USA; 5 Duke University, Durham, NC USA; 6 University California San Diego, San Diego, CA USA; 7 University of Oklahoma College of Medicine, Oklahoma City, OK USA; 8 Duke University, Durham, NC USA; 9 National Human Genome Research Institute, Bethesda, MD USA

**Keywords:** PhenX, Phenotype, Epidemiology, Risk factors, Harmonization

## Abstract

**Background:**

The purpose of this manuscript is to describe the PhenX RISING network and the site experiences in the implementation of PhenX measures into ongoing population-based genomic studies.

**Methods:**

Eighty PhenX measures were implemented across the seven PhenX RISING groups, thirty-three of which were used at more than two sites, allowing for cross-site collaboration. Each site used between four and 37 individual measures and five of the sites are validating the PhenX measures through comparison with other study measures. Self-administered and computer-based administration modes are being evaluated at several sites which required changes to the original PhenX Toolkit protocols. A network-wide data use agreement was developed to facilitate data sharing and collaboration.

**Results:**

PhenX Toolkit measures have been collected for more than 17,000 participants across the PhenX RISING network. The process of implementation provided information that was used to improve the PhenX Toolkit. The Toolkit was revised to allow researchers to select self- or interviewer administration when creating the data collection worksheets and ranges of specimens necessary to run biological assays has been added to the Toolkit.

**Conclusions:**

The PhenX RISING network has demonstrated that the PhenX Toolkit measures can be implemented successfully in ongoing genomic studies. The next step will be to conduct gene/environment studies.

## Background

The PhenX (consensus measures for Phenotypes and eXposures) Toolkit (http://www.phenxtoolkit.org) is a set of validated measures across 21 research domains that can be used to facilitate cross-study comparisons to increase statistical power to study gene/environment interactions [1,2]. The National Human Genome Research Institute (NHGRI) issued administrative supplements for the addition of PhenX measures into existing population-based genomic studies sponsored by NIH to evaluate the usefulness of the PhenX measures and to stimulate their uptake (http://grants.nih.gov/grants/guide/notice-files/NOT-HG-11-009.html). Seven research groups were funded through this granting mechanism, coming together to form the PhenX RISING (Real world Implementation and ShaRING) consortium. The purpose of this manuscript is to describe the network and the site experiences in the implementation of PhenX measures into ongoing population-based genomic studies. The information gained will be used to further improve the PhenX Toolkit and to provide guidance to other scientists seeking to incorporate PhenX measures in their studies.

## Methods

The PhenX RISING consortium comprises seven groups. A network-wide data use agreement was written and implemented to facilitate transfer of de-identified data among the seven groups and NHGRI. It is available on the PhexX Toolkit website (https://www.phenxtoolkit.org/index.php?pageLink=phenxrising). Research Triangle Institute (RTI) International (Research Triangle Park, North Carolina) serves as the administrative coordinator for the PhenX RISING network. NHGRI and RTI International documented PhenX protocol changes at each site. Monthly teleconferences between NHGRI, RTI International and the seven groups were used to share implementation findings and to discuss cross-study collaborations. Institutional certification was obtained from all sites to share de-identified data collected for this project with dbGaP (database of Genotypes and Phenotypes).

Site-specific information is summarized in Table 1. Eighty PhenX measures were implemented across the seven PhenX RISING groups, thirty-three of which were used at more than two sites, allowing for cross-site collaboration (Table 2). The PhenC Toolkit contains ID numbers for the measures and separate numbers (often ending in 1) for the detailed protocols for specific measures.

**Table 1:** PhenX RISING cohort descriptions, administration protocol and issues identified across the seven sites

**Site**	**Cohort description**	**Administration**	**Time to administer**	**Issues identified during administration/protocol changes made**
Asian Indian Diabetic Heart Study/SIKH Diabetes Study (AIDHS/SDS)	4,510 subjects aged 25+; 1,983 available with GWAS	Recruited by study staff, free health check-up and cholesterol/glucose lab results incentive; PhenX biomarker data collected using frozen sera	40-45 minutes	Not applicable
Detroit Neighborhood Health Study (Detroit)	800 subjects aged 18+ from population representative Detroit Neighborhood Health Study cohort with GWAS data	Telephone administered, $25 incentive	32.3 minutes (average)	Formatting to change instructions for telephone administration and CATI, coding, drop annual family income question due to errors
Duke University Imaging and Genetics (Duke)	200 college students and 50 adolescents. Genotyping for COMT Val Met genotype (rs4680) DAT gene (SLC6A3)	Computer-administered	60 minutes	Conversion to electronic format was difficult for some PhenX measures, but worth the investment
		Experimenter administered, data collected by computer		
Marshfield Clinic Personalized Medicine Research Project (PMRP)	3344 subjects aged 50+ from population-based biobank with GWAS data for age-related cataract, HDL, dementia, glaucoma; aged 39–100, 98% European American, 60% female	Self-administered, mailed to non-institutionalized subjects, $10 incentive	20-40 minutes	Formatting to remove instructions for person administering and scoring questionnaire, inconsistency of response order (no/yes, yes/no), skip logic errors, rules for coding
Pediatric Imaging Neurocognition and Genetics (PING)	284 subjects ages 9–21 recruited from 6 sites across the U.S. and 77 subjects from San Diego ages 3–7, all with GWAS data and neural architectural and neurocognitive phenotypes, 64% European American, 51% male	Self-administered, web-based format completed at home for retrospective cases and in the lab or at home for prospective cases, reimbursements varied by site and ranged from $20 to $40; parents of participants ages 3–7 completed the CBQ in the lab	20-90 minutes	Changed wording slightly for a few questions to make them applicable to all PING age groups, changed item orders when necessary for skip logic to work, scored short form domains using means instead of sums because of missing data
UCLA Consortium for Neuropsychiatric Phenomics	278 completed on-line protocol and 114 completed in-lab protocol; recruited from 840 eligible; ages 21–50; English speaking	Web-based questionnaires (445 questions) for on-line protocol; paper and pencil tests for in-lab protocol	60 minutes on-line; 60 minutes in-lab	Conversion to on-line administration involved some modifications to wording and logic
Chinese Longitudinal Healthy Longevity Survey (CLHLS)	In our CLHLS 2011–2012 wave, we plan to follow-up interview those CLHLS subjects aged 65+ who were interviewed in 2008–2009 wave. We have newly added 13 PhenX measures (including 32 data items) in our CLHLS 2011–2012 new wave	Home-based face-to-face interviews, with a gift of about $4. Up to middle March 2012, we have conducted interviews with 7,475 surviving CLHLS participants, and 4,914 interviews with a close family member of the deceased CLHLS Participants. So far, the response rate is 84.58%, lost-follow-up rate is 13.01%, and the refusing rate is 2.41%	About 1.5 hours	To meet the aims of this PhenX administrative Supplement project, we modified our previous CLHLS protocol by newly adding data collection of the 13 PhenX measures, including 32 data items

**Table 2:** PhenX measures being implemented across PhenX RISING sites

**PhenX ID**	**Measures**	**Detroit neighborhood health study**	**UCLA**	**PING**	**PMRP**	**AIDHS/SDS**	**Duke**	**CLHLS**	**# Groups with this measure in Common**
010100	Current age	XM	Z	X*, XM	X*, XM	Z	XM	Z	7
011000	Current educational attainment	XM	Z	Z	X*, XM	Z	XM	Z	7
010900	Current marital status	XM	Z	X*, XM	X*, XM	Z	XM	X	7
010500	Ethnicity	XM	Z	Z	X*, XM	Z	X	Z	7
010700	Gender	XM	Z	X*, XM	X*, XM	Z	XM	X	7
011300	Current employment status	XM	Z		X*, XM	Z	XM	Z	6
120500	Depression	Z	X	X*, XM	X*		Z	XM	6
010600	Race	XM	Z	Z	X*, XM	X	XM		6
030300	Alcohol - 30-day quantity and frequency	Z		XM	X*		XM	Z	5
020600	Hand dominance		Z	Z	X*, XM		Z	X	5
030800	Tobacco - 30-day quantity and frequency	Z		XM	X*		XM	Z	5
030200	Alcohol - age of first use			XM	X*, XM		X	X	4
030500	Alcohol - lifetime abuse and dependence			XM	X*, XM		X	X	4
030100	Alcohol - lifetime use			XM	X*, XM	Z	X	X	4
010200	Birthplace				X*	Z	XM	XM	4
040100	Family history of heart attack	XM			X*, XM	Z		X	4
030800	Substances - 30-day frequency	X*, XM		XM			XM	X*	4
031100	Substances - lifetime use	X*, XM	Z	XM			XM		4
030900	Tobacco - age of offset of use			XM	X*		XM	XM	4
031000	Tobacco - nicotine dependence			XM	X*, XM		X	X	4
030600	Tobacco - smoking status			XM	X*	Z	XM		4
030400	Alcohol - maximum drinks in 24 hours			XM	X*, XM		X		3
011100	Annual family income	Z		Z			Z	X	3
010400	Birthplace of grandparents				X	Z	XM		3
010800	Current address				X*, XM	Z	XM		3
060700	Current environmental tobacco smoke exposure				X*, XM	Z		X	3
180600	General self-efficacy screener		X	XM			XM		3
011500	Health insurance coverage	XM			X*, XM			X	3
130300	History of stroke - ischemic infarction and hemorrhage				X*, XM	Z		X	3
180800	Perceived stress		X	XM			XM		3
060800	Sibship/Birth order			Z	X*, XM			X	3
030700	Tobacco - age of initiation of use			XM	X*		XM		3
131000	Working memory		X	Z			Z		3
121500	ADHD symptoms		X				XM		2
060100	Characteristics of current residence				X*, XM			X	2
210200	Child-reported parental education attainment		Z				XM		2
180400	Disinhibiting behaviors/Impulsivity		X	X*, XM					2
120600	Eating disorders screener		X				X*, XM		2
180500	Emotional state		X	XM					2
131100	Executive function		X	Z					2
121300	General distress		X				XM		2
020704	Height (self-reported)				X*, XM			X	2
020703	Height (standing)					Z		X	2
120400	Hypomania/Mania symptoms		X		X				2
101100	Menstrual history						XM	Z	2
121100	Personality traits		X				Z		2
121600	Psychopathy		X				XM		2
180900	Self-esteem		X	XM					2
181000	Social isolation		X				XM		2
030900	Substances - age of first use			XM			XM		2
031400	Substances - lifetime abuse and dependence		Z	XM			N		2
120900	Symptoms of autism spectrum disorder		X				XM		2
150900	Total physical activity screener				X			XM	2
130900	Visual memory		X	Z					2
021600	Waist circumference					Z		XM	2
021502	Weight (self-reported)				X			XM	2
180100	Acculturation		X						1
120200	Anxiety disorders screener			X*, XM					1
160200	Assay for cytokine panel 12					X			1
210100	Childhood maltreatment						XM		1
100300	Contraceptive methods						XM		1
180300	Current quality of life	XM							1
141200	Fasting C-peptide assay for residual beta cell function					X			1
130700	Global mental status screener		X						1
101000	Male sexual function						XM		1
70300	Passive smoke exposure							XM	1
180700	Perceived social support/conflict		X						1
201500	Personal and family history of hearing loss							XM	1
070700	Personal perception and knowledge of smoking-related cancer risk							XM	1
211000	School social environment			X*, XM					1
141400	Serum creatinine for kidney function					X			1
101400	Sexual history						XM		1
211100	Social networks							X	1
081500	Toothache and orofacial pain							X	1
150702	Total physical activity (adult)					Z			1
150703	Total physical activity (older adult)							Z	1
061300	Ultraviolet light exposure				X*, XM				1
130800	Verbal memory		X						1
051100	Vitamin D					X			1
021501	Weight (measured)					Z			1
			12	21	26	34	4	37	35

LEGEND

X = PhenX measure.

X* = PhenX measure with changes to the protocol.

X^M^ = PhenX measure with change to mode of administration.

Z = similar data available, but not collected with PhenX.

N = Dropped.

Each site used between four and 37 individual measures and five of the sites validated the PhenX measures against other study measures (all but Asian Indian Diabetic Heart Study and Chinese Longitudinal Healthy Longevity Survey). Eight of the measures were only collected at a single site. Measures were selected to augment the data already available for the specific study cohorts and outcomes. Some sites also included additional measures to allow comparison across PhenX RISING sites. The following section contains descriptions of the seven sites, the PhenX measures employed and the administration of protocols for each site.

### The Asian Indian Diabetic Heart Study/Sikh Diabetes Study (AIDHS/SDS)

The AIDHS/SDS was established in India in 2002 and was funded by Fogarty International Center of National Institute of Health (NIH) [3]. Of the currently available 4,510 subjects from Phases I & II of the AIDHS/SDS, 1,200 subjects belong to family cohort and remaining 3,310 subjects are unrelated diabetic and healthy individuals recruited from India and the US. The goals of AIDHS/SDS are to discover unique genetic markers associated with type 2 diabetes (T2D) and related metabolic and lipid traits by performing genome-wide association scans (GWAS) and validation studies. All participants signed a written informed consent for these investigations. The AIHDS/SDS was reviewed and approved by the University of Oklahoma Health Sciences Center’s Institutional Review Board, as well as the Human Subject Protection Committees at the participating hospitals and institutes in India. Institutional certification was obtained for the submission of genotype and phenotype data of AIDHS to dbGaP.

Men and women aged 25–79 years participated. The diagnoses of T2D were confirmed by reviewing medical records for symptoms, use of medication, and measuring fasting blood glucose (FBG) levels following the guidelines of the American Diabetes Association (2004) [4], as described previously [5]. The 2 h oral glucose tolerance test (OGTT) was performed following the criteria of the World Health Organizations (WHO) (75 g oral load of glucose). BMI was calculated as (weight [kg]/height [meter] [4]). Subjects with type I diabetes, or those having a family member with type I diabetes, or rare forms of T2D sub-types (maturity onset diabetes of young [MODYs]), or secondary diabetes (from e.g. hemochromatosis, pancreatitis) were excluded from the study. The selection of controls was based on a fasting glycemia < 100.8 mg/dL (< 5.6 mmol/L) or 2 h glucose <141.0 mg/dL (< 7.8 mmol/L) were clinically free of T2D, impaired glucose tolerance (IGT).

Fasting blood samples (overnight, 12 hr) were drawn by trained assistants and serum and plasma aliquots were prepared for storage at −80°C. Blood pressure, anthropometric measurements (height, weight, and waist to hip ratio), FBG, insulin, serum cholesterol (total, HDL-C and LDL-C, and triglycerides) have been measured on all participants as described previously [5,6].

A GWAS was performed on 1,983 AIDHS/SDS subjects (980 T2D cases and 1,003 controls) from Punjabi Sikh community using a Human660W-Quad BeadChip arrays (Illumina, USA). Frozen serum samples of 1,983 subjects with GWAS data are used to perform biomarker estimations in the PhenX RISING study. We measured biomarkers related to beta cell function (c-peptide, total amylin), obesity (leptin), inflammation (TNF-α, MCP-1), T2D (vitamin D-25-OH), and kidney function (creatinine). These assays were performed following protocols and basic specifications in the PhenX Toolkit (http://www.phenxtoolkit.org/) to aid compatibility across different studies. The multiplex assays for c-peptide, leptin, total amylin, TNF- α, and MCP-1 were performed using Magnetic MILLIPLEX Human Metabolic panel from Millipore (St. Charles, Missouri) on Luminex platform (PhenX protocol #141201). The assays for 25-OH vitamin D (PhenX protocol #051100) were performed using standard monoclonal antibody-based florescence ELISA assays kits from ALPCO Diagnostics (Salem, NH). Serum creatinine was measured at Oklahoma University Medical Center Laboratory using standard Jaffe rate methodology according to the PhenX protocol (141201). All assay kits for each biomarker were used from a single source.

### Detroit Neighborhood Health Study **–** University of Michigan

The Detroit Neighborhood Health Study (DNHS) is a prospective, representative longitudinal cohort study of predominantly African American adults living in Detroit, Michigan. The overall goal of the DNHS is to identify how genetic variation, lifetime experience of stressful and traumatic events, and features of the neighborhood environment predict psychopathology and behavior. As such, the study includes two parts: a neighborhood assessment and a participant cohort. A systematic evaluation of Detroit neighborhoods was conducted June-July 2008. Data was collected on various aspects of neighborhoods, such as external building condition, sidewalk/street condition, presence of graffiti, presence of community gardens, and number of vacant lots. Cohort participants were selected with a dual-frame probability design, using telephone numbers obtained from the U.S. Postal Service Delivery Sequence Files as well as a listed-assisted random-digit-dial frame [7]. Individuals without listed landlines or telephones and individuals with only a cell phone listed were invited to participate through a postal mail effort. Participants completed a 40 minute structured telephone interview annually between 2008–2012 to assess perceptions of participants’ neighborhoods, mental and physical health status, social support, exposure to traumatic events, and alcohol and tobacco use; each participant was compensated $25USD [7,8]. All survey participants were offered the opportunity to provide a blood specimen (venipuncture, blood spot, or saliva) for immune and inflammatory marker testing as well as genetic testing of DNA [9]. Participants received an additional $25USD if they elected to give a sample. Informed consent was obtained at the beginning of each interview and again at specimen collection. The Institutional Review Board of the University of Michigan reviewed and approved the study protocol.

Of the 1,547 participants in Wave 1 (Sept. 2008- April 2009) and Wave 2 (Sept. 2009- June 2010), 917 provided a biospecimen sample yielding DNA; 800 were randomly selected for GWAS testing using the Illumina OmniExpress GWAS chip. The fourth wave of the DNHS interview incorporated PhenX Toolkit phenotype measures (Table 2) and targeted these 800 individuals. Institutional certification was obtained for the deposition of genetic and phenotypic data into dbGaP.

### Identifying and modifying a risk phenotype for self-regulation failure - Duke University

The parent study was designed to validate a hypothesized gene/environment/self-regulation risk phenotype (a combination of individual differences in regulatory focus, COMT genotype, and chronic failure to attain a particular kind of personal goal) that is believed to confer vulnerability to failures of self-regulation, which in turn increase risk for psychopathology with significant public health implications such as aggression, gambling, and excessive use of alcohol and other drugs. The parent study includes the best-validated measures in the field of imaging genetics for quantifying the phenotypes of interest. This list shares little overlap with the specific measures included in the PhenX toolkit, but significant overlap in the domains of interest including Psychiatric, Psychosocial, and Alcohol, Tobacco, and Other Substances. Conceptually overlapping measures from these domains were integrated into our existing protocol, allowing for evaluation of relationships between PhenX toolkit measures and behavioral, clinical, neural, hormonal and genetic variables of clear significance for psychopathology. Thus, the broad goals of our proposed research are (1) to add measures from the PhenX toolkit that overlap with phenotypic measures in the existing study and (2) to add measures from the PhenX toolkit that represent important areas of measurement that were not included in the parent grant because they were not specifically related to the aims of that investigation. We will then evaluate the utility of PhenX toolkit measures on several criteria including validation against intermediate neurobiological phenotypes identified through functional neuroimaging.

Over the 1-year funding period, we collected data on N = 200 subjects from a college sample and N = 50 subjects from an adolescent sample. All subjects were recruited through existing protocols approved by the Duke University Medical Center Institutional Review Board and provide written informed consent before participation. We will now work with collaborators to combine our data sets with others that have used the same PhenX tools to provide the greatest power possible to address questions of genetic influences on phenotypes of interest to our colleagues in the field as well as those phenotypes most directly related to our own work. Of note, we anticipate continuing to use the added PhenX measures for the duration of the parent project, giving a total sample size of N = 400 college students and N = 100 adolescents.

### Marshfield Clinic Personalized Medicine Research Project (PMRP)

The Marshfield Clinic Personalized Medicine Research Project (PMRP) is a population-based biobank linked to the electronic health records of Marshfield Clinic [10]. After providing written informed consent, subjects aged 18 years and older completed questionnaires that included questions on demographics, family health history, smoking and alcohol exposure and dietary intake [11] and physical activity questionnaires. The biobank was reviewed and approved by the institutional review board (IRB) of Marshfield Clinic. The PhenX RISING project was reviewed and approved by the IRBs at Essentia Institute of Rural Health, Marshfield Clinic and Pennsylvania State University.

The Marshfield Clinic PMRP is a member of the NHGRI-funded eMERGE network (http://www.gwas.net)[12]. The goal of eMERGE1 was to conduct genome-wide association studies using electronic health records to define phenotype. The primary Marshfield phenotypic outcomes used to identify subjects for GWAS genotyping were age-related cataract and HDL. Additional subjects were genotyped for dementia, resistant hypertension and open-angle glaucoma. The subjects with GWAS data who were alive with known, non-institutionalized addresses and who had given consent for re-contact were eligible for participation in the PhenX RISING study.

The PhenX measures listed in Table 2 from the PhenX Toolkit were incorporated into a 32-page, self-administered questionnaire. The questionnaires were mailed with a cover letter to eligible subjects with a stamped, self-addressed envelope. A second mailing was employed to maximize the response rate. Subjects were offered $10 for their time to complete the questionnaire. PhenX responses were validated using data from PMRP questionnaires and Marshfield Clinic electronic health records [13].

### Pediatric Imaging, Neurocognition, and Genetics (PING)

Pediatric Imaging, Neurocognition, and Genetics (PING) is a multi-site cross-sectional study of typically developing children, adolescents, and young adults ranging in age from 3 to 20 (see Acknowledgements for a description of participating members from the PING infrastructure) funded by the National Institute on Drug Abuse (NIDA) and the National Institute of Child Health and Human Development (NICHD). The primary goal of PING is to create a pediatric imaging-genomics database of approximately 1400 cases that is freely available to the scientific community. Participants aged 18 and up provided written informed consent to undergo approximately three hours of neurocognitive testing and a one hour neuroimaging session, and to provide a saliva sample for [14,15]. The majority of participants also consented to allow these data to be shared in the publicly available database. For participants under the age of 18, parent versions of this consent were signed and the children and adolescents provided their assent where appropriate. This study structure was approved by IRBs at all participating PING sites. Six of the 9 PING sites chose to participate in the PhenX RISING project, and each participating site’s IRB approved this project as well.

Initially, only self-report PhenX measures were chosen for inclusion in PING. Given the reading limitations of the youngest children in the PING age range, only participants ages 9 and above were asked to complete these measures. Although the original PING age range was 3 to 20, a few of the participants were 22 years old by the time they were brought back to complete the PhenX measures. Between the 6 sites who opted to participate in the PhenX RISING project, 585 subjects met the initial age criteria for inclusion. Subsequently, the UC San Diego site opted to include data from one PhenX parent-report measure (Childhood Behavior Questionnaire; CBQ) that they were already administering in the lab for 3 to 7 year old participants prior to beginning the PhenX RISING project. Table 2 lists all the PhenX measures that were chosen for inclusion. Not all of the original measures were deemed appropriate for all ages. As such, study arms were created for different age ranges, and measures were included in each age range as appropriate. Table 3 indicates which measures were used in each study arm. Several PhenX instruments were available in both child/adolescent and adult versions. With one exception, child/adolescent versions were used for all participants in order to maximize consistency across PING cases. Separate versions were used only for the General Self-Efficacy scale because the child version asked a large number of school-related questions that were not appropriate for young adults. Some questions were modified from their original form in order to broaden their applicability to all participants within the PING age range (see Table 4 for specific modifications).

**Table 3:** PhenX measures being implemented in PING by study arm

	**Study arm (age range)**				
**PhenX toolkit measure**	**9-10**	**11**	**12-13**	**14-17**	**18-21**
Demographics - age (#010101)	✔	✔	✔	✔	✔
Demographics - gender (#010701)	✔	✔	✔	✔	✔
Demographics - marital status (#010901)					✔
Emotional state, child protocol (#180502)	✔	✔	✔	✔	✔
Depression, child protocol (#120501)	✔	✔	✔	✔	✔
Anxiety disorders screener, child protocol (#120201)	✔	✔	✔	✔	✔
Disinhibiting behaviors - impulsivity, child protocol (#180402)		✔	✔	✔	✔
General self-efficacy, adult protocol (#180601)					✔
General self-efficacy, child protocol (#180602)		✔	✔	✔	
School social environment (#211001)		✔	✔	✔	✔
Tobacco - 30-day quantity and frequency (#030801)			✔	✔	✔
Tobacco - age of initiation of use (#030701)			✔	✔	✔
Tobacco - age of offset of use (#030901)			✔	✔	✔
Tobacco - smoking status (#030601)			✔	✔	✔
Tobacco - nicotine dependence (#031001)			✔	✔	✔
Perceived stress (#180801)				✔	✔
Self-esteem (#180901)				✔	✔
Alcohol - 30-day quantity and frequency (#030301)				✔	✔
Alcohol - age of first use (#030201)				✔	✔
Alcohol - lifetime abuse and dependence (#030501)				✔	✔
Alcohol - lifetime use (#030101)				✔	✔
Alcohol - maximum drinks in 24 hours (#030401)				✔	✔
Substances - age of first use (#031201)				✔	✔
Substances - lifetime abuse and dependence (#031401)				✔	✔
Substances - lifetime use (#031101)				✔	✔

**Table 4:** PhenX protocol changes implemented by sites

**PhenX ID**	**Description of protocol change**	**Group**
120600	Had to add ‘0’ option for how many times the person throws up because there was no skip logic	Duke
030300	Did not modify “Protocol Text” field but modified other fields (frequency, used “everday” instead of “every 30 days”)	CLHLS
010100	Did modify “Protocol Text” field for specific applications (added “animal year” for birth year)	CLHLS
011000	Did modify “Protocol Text” field for specific applications (added “the year of attending school” instead of the degree of education)	CLHLS
011300	Did modify “Protocol Text” field for specific applications (most of respondents are retired at present)	CLHLS
010500	Did modify “Protocol Text” field for specific applications (respondents are Chinese, not Americans)	CLHLS
101100	Did not modify any fields but used a subset of the protocols	CLHLS
030800	Did not modify “Protocol Text” field but modified other fields (frequency, used “everday” instead of “every 30 days”)	CLHLS
150703	Did modify “Protocol Text” field for specific applications (added “playing mah-jong”)	CLHLS
011100	Unfortunately, we had to drop the PhenX family income measure. If the participant does not outright answer their best estimate for total family income, a series of higher or lower questions are asked which relies on poverty threshold information determined by the US Census Bureau. However, the poverty threshold levels contradict the pre-determined series of higher or lower questions such that a family could be making more than $30 K a year but still be under the poverty threshold depending on the number of members living in the household. Thus, we felt this question was subject to error and we decided to revert to the family income question asked in previous waves of our survey. (See attached word document)	Detroit Neighborhood Health Study
031100	For the Substance use questions, we decided in conjunction with our survey company to alter these questions for administration simplicity, while ultimately still obtaining the answers for the PhenX questions. First, we ask about ANY use of the 10 categories of substances for both lifetime and 30 day frequency. Then, we ask about ILLICIT use for lifetime and 30 day frequency.	Detroit Neighborhood Health Study
030800	For the Substance use questions, we decided in conjunction with our survey company to alter these questions for administration simplicity, while ultimately still obtaining the answers for the PhenX questions. First, we ask about ANY use of the 10 categories of substances for both lifetime and 30 day frequency. Then, we ask about ILLICIT use for lifetime and 30 day frequency.	Detroit Neighborhood Health Study
010100	Changed “refused” response option to “decline to state”	PING
010700	Changed “refused” response option to “decline to state”	PING
010900	Changed “refused” response option to “decline to state”	PING
180400	Did not modify any protocol text, but modified the age range such that the child version will be given to all participants (up to age 21)	PING
211000	Added instructions and a question at the beginning asking for an education level so that the questionnaire could be administered to only the participants who are still in school. Also changed wording of items 7 (“help us children with our…” to “help students with their…”), 9 (“teacher” to “teachers”), and 37 (“when we play” to “when we do activities”) in order to make it apply to entire age range.	PING
120500	Administering the child protocol to all participants ( up to age 21). Also modified wording of items 4 (“other kids” to “others”) and 15 (“kids” to “people”) in order to make it applicable to entire age range.	PING
120200	Administering child version to all participants (up to age 21). Changed the wording of items 6 (“I want that things are in a fixed order” to “I want things to be in a fixed order”), 52 (“I worry that bad happens to my parents” to “I worry that bad things happen to my parents”), and 64 (“I have unbidden thoughts about a very aversive event I once experienced” to “I have unwanted thoughts about a very unpleasant event I once experienced”) in order to make them more easily understood by young children.	PING
All Alcohol, Tobacco, & Substance Questionnaires	Administered all questions, but added questions and modified order when necessary to allow for the skip logic to work properly in Assessment Center. Will be emailing reference cards as separate files in email to participants.	PING
180500	Did not modify any protocol text, but modified the age range such that the child version will be given to all participants (up to age 21)	PING
030300	Removed don’t know/refused option and from [DATEFILL] not used, cards not used	PMRP
030200	No changes, definition/cards not given	PMRP
030100	No changes, definition/cards not given	PMRP
030500	No don’t know/refused option, did not include question 3 as had just answered largest number of drinks in a single day, did not use skip for 3 or fewer drinks or questions 1 and 2 never, just continued with tolerant questions, included card E2 for them to determine 50% increase or not, put YES as first option to keep consistent throughout, removed tally sheet references, did not include question 5.2a as no don’t know option given, question 10 put Did drinking ever cause you to have into table same with question 12 lead in, 13.12 which ones removed from table and just asked them to specify which ones	PMRP
030400	Didn’t give definition or flashcards for size of a drink	PMRP
010200	Added a Don’t Know option	PMRP
010300	No changes	PMRP
010400	No changes	PMRP
060100	Added Your to Type & Age of Home, did not use 5. when did you move from there?, current pets removed refused/don’t know option,	PMRP
010800	Changed from verify address to list your complete address	PMRP
010100	Removed don’t know/refused option and about how old are you	PMRP
011000	Removed refused option	PMRP
010900	Removed don’t know/refused options	PMRP
011300	Changed what in the question to other to match the selections	PMRP
060700	To question 1 added in your current household, removed the refused/don’t know options. For who smokes created a table to complete	PMRP
120500	Removed from the Composite International Diagnostic Interview from the section header, removed refused/not asked options, if question 4 was yes, asked 4a didn’t skip to 5 as direction indicated, if no skip to 5, Part II removed scoring	PMRP
010500	Removed refused don’t know from first question and refused from second question	PMRP
040100	Added 2 more lines for sister, brother, daughter, son	PMRP
010700	Removed refused/don’t know options	PMRP
020600	Added Which hand do you use to: to the table, added only to the right hand and left hand column header	PMRP
011500	Removed refused option, added our Badger Care	PMRP
020704	Removed refused/don’t know options, listed as ____FEET ____INCHES	PMRP
130300	Started A. at SUDDEN LOSS OR CHANGE OF SPEECH, removed don’t know from question 3, 5e had question read If more than one problem with you speech, which of these most closely describes the problem with your speech?, in 6 removed INCLUDE ALL THAT APPLY and READ ALL directions, I THINK INCORRECTLY CHANGED SKIP IN 9 SHOULD HAVE ASKED 9A FOR BOTH EYES AND ASKED FOR RIGHT AND LEFT INSTEAD, in 10 removed INCLUDE ALL THAT APPLY, 11 11a 14 15 removed don’t know,	PMRP
011400	Made into a table and added space for one more person	PMRP
120400	No changes	PMRP
010600	Removed refused option	PMRP
060800	Changed I to we, bolded ask about your full brothers and sisters, removed NA option in question 2, to 3 added List oldest to youngest, putting yourself in the birth order, changed name to initials, removed NA option, last column reads How old are they now/were they when he/she died	PMRP
030900	Removed don’t know/refused option	PMRP
030600	Removed don’t know refused options, removed skip after question 2, if question 1 no went to smoke exposure section,	PMRP
031000	But b. at the end of the 6 questions with direction to return to answer for max use if appropriate,	PMRP
030800	Changed directions to read The following are three sections. Section A is for Every-Day Smokers. Section B is people who are Some-Day Smokers. Section is for Former Smokers. Removed don’t know/refused options.	PMRP
030700	Removed skip directions and asked both question of all smokers, removed don’t know/refused options	PMRP
150900	Removed scoring table	PMRP
061300	Added don’t know option to direct to question if don’t know exact age question, and reworded the ps in the table to read on a typical weekday in the summer, on a typical weekend day in the summer, For how many months a year did you usually have a tan, row ps read when you were in your (teens or twenties or thirties or the past ten years) about how many hours did you generally spend in the mid-day sun.	PMRP
021502	Requested in lbs.	PMRP

Although PING recruitment strategies varied by site, the general approach that was taken was to enroll and complete participants in the older age ranges first. This strategy allowed investigators to observe responses to testing and imaging, and to better anticipate and plan for any challenges that seemed likely to arise when running younger subjects. PhenX Toolkit measures were not incorporated into PING data acquisition protocols until most children over age 8 were already completed. As such, the time between collection of the initial PING deliverables and the collection of PhenX data varied greatly for participants who were enrolled in PING after the addition of the PhenX protocol. Overall, the time difference ranged from 0 to 2.5 years (M = 0.93, SD = 0.76). Participants who were adolescents when they assented to participation in PING, but then turned 18 prior to PhenX completion, were asked to complete an additional adult consent form.

In order to improve response rate from participants who already completed PING, it was decided that PhenX data would be completed in a web-based format. An NIH-sponsored web-based data collection tool called Assessment Center (http://www.assessmentcenter.net) was used for online data collection. A multi-arm study was created in Assessment Center, with each arm representing an age range, and the PhenX instruments were added to the study arms as appropriate. Items appeared on the screen one at a time, and participants could choose a response option or press “Next” to skip to the next question. Participants could also click “Previous” to go back and change a response to a previous item within an instrument. The structure of the alcohol and substance abuse instruments was changed slightly, and skip logic was employed, in order to adapt them to the web-based format.

Participant recruitment and reimbursement strategies for the PhenX RISING project varied by PING site. For retrospectively collected cases, some sites called or emailed participants or parents and offered an opportunity to participate in exchange for reimbursement. Other sites brought participants back into the lab for additional studies, and asked them to complete the web-based PhenX study at that time. Sites also differed on prospective data collection procedures, where some collected PhenX data in lab and others allowed participants to complete the questionnaire from home. When login information was emailed to participants, a username was sent in an email with the study link, and a password was sent in a separate email for security purposes. Reimbursements were sent after verification of completion, and ranged from $20 to $40.

### UCLA Consortium for Neuropsychiatric Phenomics

The Consortium for Neuropsychiatric Phenomics comprises eight linked grants awarded under the aegis of the NIH Roadmap Initiative. The PhenX supplement grant was awarded to the Human Translational Applications Core, a center core that conducted extensive phenotyping of more than 1000 healthy volunteers aged 21 to 50 in the Los Angeles metropolitan area from 2007 to 2012. The phenotyping efforts focused on two primary themes – memory mechanisms and response inhibition mechanisms – and participants completed approximately 12 hours of cognitive phenotyping, and a subset of these participants received also several hours of neuroimaging procedures to examine brain structure and function (descriptions of these procedures are available at http://www.phenomics.ucla.edu). The PhenX supplement to this protocol focused on behavioral and cognitive variables, and involved two components: (1) a Web-based component comprising participant self-report questionnaires, which was offered to all English-speaking completers of the parent study who agreed to be recontacted; and (2) an in-laboratory study English-speaking completers who were willing to have additional procedures conducted in the laboratory. These measures are listed in Table 2. Participants received $15/hour for participating and those who came for in-lab procedures additional received reimbursement for public transportation or parking. The project was approved by the UCLA IRB.

### Chinese Longitudinal Healthy Longevity Survey (CLHLS) site

To gain better understanding of social, behavioral and genetic factors and their interactions may affect healthy longevity, as well as to provide database for academic research, health and aging policy analysis, the Chinese Longitudinal Healthy Longevity Survey (CLHLS) conducted about 80,000 face-to-face interviews with participants in 1998, 2000, 20002, 2005, and 2008/2009, respectively. Among about 80,000 interviews conducted in the CLHLS 1998-2008/2009 five waves, 14,376 interviews were with centenarians, 18,938 with nonagenarians, 20,823 with octogenarians, 14,285 with young-old aged 65–79, and 10,962 with middle-age adults aged 35–64. Data on mortality and health status before dying for the 17,649 elders aged 65–110 who died between follow-up waves were collected in interviews with a close family member of the deceased. The survey areas covered 22 provinces sharing 85 percent of total population in China. The CLHLS datasets have been publicly available and widely applied in aging studies by scholars around the world.

In the CLHLS 1998 baseline survey and the 2008/2009 5^th^ wave, DNA (blood and saliva) samples were collected from 18,093 interviewees, including 3,193 centenarians, 4,821 nonagenarians, 4,076 octogenarians, 3,441 young-old aged 65–79, and 2,619 adults aged 40–64.

Supported by the administrative supplement awarded by The National Human Genome Research Institute, the CLHLS team added 13 PhenX measures (including 32 data items) in our CLHLS 2011/2012 new wave. These additional relevant standard phenotypic and environmental exposure measures related to healthy aging selected from the NIH PhenX Toolkit will be used together with other internationally-standardized data which have been collected in CLHLS to address scientific questions on the effects of genetic, social, behavioral, environmental factors and their interactions on healthy aging at old ages.

The CLHLS study protocols (such as the informed consensus forms and other relevant materials) was reviewed and approved by the Institutional Review Boards of Duke University and Peking University.

In addition to the site-specific projects outlined, cross-network analyses are being undertaken for three projects where two or more sites have collected the same PhenX measures. Data harmonization for race/ethnicity was undertaken across all seven sites. This measure was chosen because it was being used by all sites and several sites had more than one measure used. Also, race/ethnicity is important for gene/environment analyses and the administrative supplement was specifically made available to support gene/environment studies. The process employed to harmonize the measures was first to compare the questions asked and the mode of administration. The PhenX Toolkit measures were considered to be the common measure for harmonization. The next step was to determine if the race/ethnicity categories were the same for all sites. The category “other” served as the common denominator where sites did not have the same level of detail. Finally, variable names and codes were checked for consistency.

## Results

Study-specific protocols and data will eventually be available in dbGaP for all of the PhenX RISING sites.

The various implementation strategies employed across the sites and different study populations resulted in different response rates and knowledge gained (Table 4). Site-specific experiences follow.

### Asian Indian Diabetic Heart Study/Sikh Diabetes Study (AIDHS/SDS)

Quantification of serum biomarkers using PhenX Toolkit measures were performed on frozen serum samples of participants with genotyping data available from GWAS. Informed consent was obtained from each individual upon initial inclusion into these investigations for participation in genetic and biomarkers study therefore no additional contact was required for these investigations. Results for each biomarker were included in an extensive database for analysis. Enrichment of GWAS data with additional biomarkers could lead to identification of variants regulating important metabolic pathways through cross-study analysis.

Two planned assays were not run because they would have used too much of the remaining biological sample. After discussion with RTI, ranges of sample volume requirements was added to the PhenX Toolkit.

Our study strongly recommends biomarker assay optimization (especially those measured using different platforms) to reduce inter-study variability.

### Detroit Neighborhood Health Study

The PhenX Toolkit measures were incorporated into the fourth interview wave of the DNHS. The PhenX Toolkit measures required formatting for telephone administration and CATI programing from their original written application. Questions were re-numbered to fit into the existing annual survey structure. Response coding was adjusted to match existing survey codes for consistency.

The PhenX Toolkit measure for Annual Family Income was not included in the final version of the survey. The Annual Family Income measure requires the interviewer to have information on current poverty levels from the U.S. Census Bureau. We found the question structure was not compatible with certain poverty threshold scenarios based on 2008 poverty data for Detroit, MI. For example, it would be possible for a participant to have an annual family income above $35,000, yet still be below the poverty threshold based on their family size. As a consequence, their response would not trigger the poverty threshold specific income component of the PhenX question because they fell into a previously asked income category. Due to this inconsistency and potential for incorrect classification, we reverted to an annual family income question structure successfully implemented in previous survey waves.

We also found it necessary to change the administration of substance use questions for both the lifetime use and 30-day frequency. These questions were originally developed to be asked at an in-person interview and the materials on the PhenX Toolkit include a “flashcard” describing the various types of substances included in this measure. To effectively adapt these questions for telephone administration and determine licit from illicit use, we modified the structure to ask: 1- if the participant had ever used the substance in their lifetime, 2- if the participant answered yes to #1 they were asked the 30-day frequency of use, 3- if the participant answered yes to #1, they were asked if they ever used the substance illicitly in their lifetime for the drug categories sedatives, tranquilizers, painkillers, stimulants, and marijuana, 4-if the participant answered yes to #3, they were asked the 30-day frequency of illicit use, again for the drug categories sedatives, tranquilizers, painkillers, stimulants, and marijuana. All reported use of the drug categories cocaine, hallucinogens, inhalants/solvents, and heroin was assumed to be illicit as they are controlled substances. These alterations kept the essence of original measure yet tease out the difference between licit and illicit use in a telephone interview format.

The survey was administered to participants by Abt SRBI (New York, NY) beginning in September 2011 and concluded in February 2012. The average administration length was 32.3 minutes and a response rate of 80% (845 of 1050) was achieved.

The Aiello Group identified some limitations associated with a few PhenX toolkit measures in their survey when applied to their population of participants in the “Detroit Neighborhood Health Study”. Certain validated measures, such as substance use and annual family income, had to be altered to be successfully administered by telephone in the DNHS population. Though the Aiello Group supports the use of standardized measures to foster collaboration and analysis between studies, further refinement of the PhenX Toolkit measures will be needed to reflect the diverse settings in which they may be used, such as phone, personal computer, or in-person interviews.

### Duke University Imaging Genetics Study

The response rate for PhenX measures in this study was 100%. The high response rate was likely due to the conditions of the study. Participants required minimal additional instruction from research staff, suggesting that online administration of the PhenX measures is viable.

The PhenX Toolkit measures required time to format for computerized administration, including automated skip-logic (i.e., creating computerized instructions to ignore some questions if previous answers suggest they are irrelevant) and custom formatting of some items. This initial investment of resources resulted in significant advantages over paper administration however once the questionnaires were converted to electronic format. The value of computerized administration increases with sample size, such that in any large scale study, it is difficult to imagine using a paper format unless absolutely required. We would like to emphasize however that PhenX measures requiring an interviewer were generally avoided for this study and would pose unique challenges to computerized administration.

### Marshfield Clinic Personalized Medicine Research Project

The strategy of two mailings with a modest financial incentive has been used successfully in Marshfield previously for self-administered questionnaires [11]. With the 32-page PhenX self-administered questionnaire, this strategy resulted in a 70% response rate.

The PhenX Toolkit measures required substantial time to format for self-administration (Table 4). The instructions for a person to administer the questions and the instructions for scoring some of the sections had to be removed. Distracting notations for data entry were deleted. Response order was changed to be consistent between questions, such that “yes” always came before “no”. “Refused” was deleted as a response category. Numbering was changed to reflect the total number of questions included. RTI responded by creating an option to select “self-administered” within the PhenX toolkit when creating the data worksheets.

A number of rules for coding non-standard responses were developed and shared with RTI International and NHGRI program staff to be incorporated into the Phenx Toolkit. Where subjects entered numbers that were not integers and the PhenX measure only allowed for integers, numbers were rounded up. When subjects indicated two education levels, the highest was selected for data entry. Improbable responses such as a height of 11 feet were changed to missing data. In the section with questions about health problems related to drinking, if a health problem not clearly related to drinking (such as a hiatal hernia) was indicated, that response was not used. In the depression symptom assessment, if more than one response category was entered the more severe level was entered. If number ranges were given, the mid-point of the range was entered and rounded up if an integer was required.

In the data cleaning process, several genders errors were discovered. After checking the medical records for these subjects, it became clear that the spouse of the intended subject had completed the PhenX questionnaire.

One of the biggest issues I think we had discussed in the beginning was the way the Domains had to be transferred from their original form to the questionnaire form. (very time and labor intensive).

The Domains could have been written in a more basic easy to follow manner and then ready to be inserted in to questionnaire form. To be very explicit and try to eliminate the replies that result in such outliers.

Scoring was huge issue. This again should be more consistent across all the sites. Our site overcame the inconsistent answers by looking at each of the situations individually and creating a rule for each situation.

### Pediatric Imaging, Neurocognition and Genetics (PING)

As previously noted, strategies for acquiring data from participants who already completed the rest of their PING assessment varied by site. As such, response rates also varied by site. Of the original 585 participants targeted at the 6 participating PING sites, 286 completed the PhenX measures (49%). The UC San Diego site added 77 CBQ parent-report measures in the 3 to 7 age range, for a total 361 cases (2 participants who completed the CBQ at age 7 were also among the participants who completed the web-based assessment when they turned 9). The length of the online study varied according to the age-based study arm the participant qualified for, but completion time ranged from 20 minutes for children ages 9–10 to 1.5 hours for adolescents who endorsed use of a variety of substances.

Creation of the study in Assessment Center had a number of strengths and weaknesses. Assessment Center was designed for the purpose of secure data collection, and this made it an ideal medium for collecting this small amount of data and adding it to the larger set of data that was already collected for these participants. Creation of the short form instruments was simple, and once an instrument was created, it could be placed in as many study arms as necessary, or even shared with other studies in Assessment Center. Creation of the substance use forms was somewhat more difficult. The skip logic options in Assessment Center are relatively basic, allowing the instrument only to skip ahead on the basis of specific responses to the current item. It would not allow for more complex branching involving decisions based on responses to previous questions. Therefore, it was sometimes necessary to change the order of items or add additional items to allow the instrument to flow continuously from beginning to end. In addition, some of the substance use questionnaires came with reference materials describing alcoholic beverages and substances for the participant. Such materials could not be provided on-screen with the relevant questions using Assessment Center. Because of this, a PDF file was created with the reference materials and emailed to the relevant participants when sending them the study link.

For the purpose of ongoing quality assurance, data were scored using the PhenX protocol. One issue that was discovered relating to online data collection was that some items were skipped. Items that were skipped by design due to the program’s skip logic were denoted by missing values in the output table. However, skipped items for which a response was expected were denoted with the word “SKIP” in the output. When a participant skipped an item, there was no way of knowing if this was accidental or whether s/he chose to skip it, and if so, why. Some may have skipped items because they did not feel comfortable answering, but others may have skipped because they did not understand the question. This may be one potential drawback to collecting data online rather than in a lab where a researcher can answer any questions and ensure that any missing data was intentional and/or unavoidable. Because most of the scoring instructions for the PhenX short form measures involve summing items across subdomains, missing items heavily impacted scores. As such, it may be necessary to develop scoring protocols that either compute mean scores rather than summed scores, or impute missing data.

A number of challenges arose in the implementation of the PhenX RISING supplement to the PING study. Because PING is itself a multisite initiative taking place at 9 different sites across the U.S., we ran into a number of problems throughout the data collection and sharing process. Each site had specific language regarding what measures would be administered and how that data could be shared in their consent forms and IRB protocols, and this language was not standardized across sites. Because the measures given through the PhenX RISING initiative were added on to an already existing protocol that was specific to each site, the process of amending IRB protocols was time consuming. It turned out that it was not feasible for each site to amend its IRB to accommodate the collection of PhenX RISING data. As a result, three of our nine sites opted not to participate in PhenX RISING. Additionally, another two sites determined after data collection was complete that they were unable to share the data that was collected. Because of this, we would strongly recommend that multisite studies standardization their IRB protocols as early as possible, paying particularly close attention to data sharing language.

Another challenge associated with PhenX data collection was that a large number of PING participants had already completed their visit before the PhenX initiative was implemented. As a result, we felt that the best chance we had of maximizing our response rate to PhenX measures was to offer the battery of questionnaires as an online survey. We spent a great deal of time converting PhenX measures to Assessment Center, and we ran into a number of problems with questionnaires that used loops and skip logic. Overcoming these obstacles took some time. This barrier and the IRB difficulties described above were the primary reasons why so many PING participants had participant had completed their visits by the time we were able to launch PhenX data collection. Despite our efforts to make it as easy as possible to respond to our PhenX questionnaires, we ultimately overestimated the number of participants from which we would be able to acquire the added PhenX data. If we had our IRB issues resolved earlier, it would have been very useful to have an already existing web-based mechanism for acquiring data. One way of accomplishing this would be to have standard versions of these questionnaires in Assessment Center.

In addition to the challenges described above, we also learned a valuable lesson about the use of these measures in a developmental sample. The PhenX Toolkit has a number of measures that have child and adult versions, and this is useful for studies with more narrow age ranges. However, our sample ranged in age from 3 to 20. We were not able to find measures that could be given across our entire age range. As a result, we ended up with much smaller sample sizes than we had hoped for many of the measures, even when taking the other challenges we faced into consideration. Combining versions was often not possible because the administration format and domain scores are often quite different. We attempted to get around this to some extent by modifying the wording of some questions so we could expand the age range of a single form. However, to the extent possible, it would be very helpful if some measures could be identified for inclusion that span a wider age range for children and adolescents.

### UCLA Consortium for Neuropsychiatric Phenomics

UCLA Consortium for Neuropsychiatric Phenomics Paralleling other sites we found that the formatting of PhenX measures to our unique Web-based platform involved more effort than we would have hoped, particularly for certain branching questionnaires. Given the high likelihood that future studies may well move towards increasing Web-based data acquisition, it may be useful to consider developing a centralized Web-service that would help better standardize the acquisition process and data capture, because the current model is going to involve yet another “translation” to integrate with other PhenX data even though the data are designed to be compatible.

For the in-laboratory components of examination (in our case, for neurocognitive phenotyping), we think standardization would be enhanced if PhenX were to provide standard instructions and training guidelines. Our group has extensive experience with the PhenX instruments, but as we organized the training it became clear that there are many “devils in the details” of test administration training and quality assurance that we are familiar with as a site primarily dedicated to cognitive assessment, but sites with less experience aiming to “add on” some cognitive phenotype measures will likely benefit from more guidance. For example, the different vendors of the psychological tests do not uniformly provide instructions on key elements of the examination procedure including stimulus presentation, response collection, and scoring of ambiguous responses.

### Chinese Longitudinal Healthy Longevity Survey (CLHLS)

The data entry and cleaning for the CLHLS 2011 survey in all other sampled areas was completed. We have conducted interviews with 7,375 surviving CLHLS participants aged 65+, and 4,918 interviews with a close family member of the deceased CLHLS Participants aged 65+. The response rate of our CLHLS 2011 survey is 86.1%, lost-follow-up rate (mainly due to outmigration and the interviewers could not find them any more) is 11.5%, and the refusing rate is 2.4%. The refusal rate was fairly close to that in previous waves, which may show that the newly added 13 PhenX measures (with 32 data items) are in general workable among Chinese elderly population. The interview refusal rate among the Chinese elderly especially the oldest-old was low. The low refusal rate likely is due to the fact that the Chinese elders especially the oldest-old in general like to talk to outside people, plus they stay at home without a job or other duties. Many of the oldest-old and their family members may also feel honored to participate in survey interviews concerning healthy longevity, as they may be proud of being a member of a long-lived group.

Age reporting of Han Chinese consisting of 94.4% of the total sample of our CLHLS 2011 survey is acceptably accurate, which is rather unique as compared to many other developing countries. Acceptably accurate age reporting among the Han Chinese elderly including the oldest-old is due to their cultural tradition of memorizing their date of birth for determining important life events such as dates of engagement, marriage, starting to build a residential house, and even for long-distance traveling. This has been confirmed previously [16]. We have conducted evaluations of the data quality of the CLHLS 2011 survey including the newly added PhenX measures. The evaluation include assessments of mortality rate, proxy use, non-response rate, sample attrition, reliability and validity of major health measures, and the rates of logically inconsistent answers, with generally satisfactory results compared to other major aging studies. Factor analyses on cognitive functioning, physical performance, and functional limitations demonstrate that the interviewees’ answers to questions concerning different aspects of the same category are generally consistent. The rates of logically inconsistent answers and incomplete data are relatively low. Careful assessments have led us to believe that, similar to previous CLHLS waves, the data quality of the CLHLS 2011 survey is generally good. However, we realize that some problems also exist in the datasets, which will be addressed in our forthcoming technical reports.

As the first batch of the results of our CLHLS PhenX study component, we have produced a 35-page report including 34 tables containing the 34 data items in the 2011 CLHLS questionnaires corresponding to the 13 newly-added PhenX measures, supported by the NIH administrative supplement grant awarded to CLHLS research team. These PhenX measures are based on the healthy-ageing relevant items from the internationally well-known PhenX Toolkit (https://www.phenxtoolkit.org/), and adopted to Chinese culture and social reality.

As previously planned, our CLHLS 2012 survey in 8 longevity areas (counties or cities) where the density of centenarians is exceptionally high is still ongoing. We adopts the same study protocol but with added more sophistic components in our 2012 survey in these 8 longevity areas, as compared to the survey in the other sampled areas of the 22 provinces surveyed in 2011. We expect to complete all field work of face-to-face interviews around the end of October in 2012 (note: Our previous fifth wave of CLHLS was conducted in 2008/2009, and thus the current sixth wave is in 2011/2012). We will conduct data analysis on the relevant PhenX measures newly collected, aiming at: (1) to enhance the interdisciplinary research of genetics and its interactions with social and behavioral factors; (2) to broaden the scope of our CLHLS study and combine it with other investigations using the same or similar PhenX measures to increase power and efficiency of discoveries on effects of genetic, social, behavioral factors and their interactions on healthy aging.

As demonstrated in Table 5 by the large number of study subjects representing diverse racial/ethnic groups, the PhenX RISING network was able to successfully implement PhenX measures into ongoing studies in a relatively short time frame (one-year administrative supplements to parent grants).

**Table 5:** Demographic data by site

**Group**	**Sample size**	**Median age and range**	**# Male**	**Male median age and range**	**Female median age and range**	**African American**	**White**	**Asian**	**American Indian**	**Native Hawaiian**	**More than one race**	**Hispanic**
Asian Indian Diabetic Heart Study/SIKH Diabetes Study (AIDHS/SDS)	1782	53	936	53	52	0	0	1782	0	0	0	0
		19-89		19-86	20-89							
Chinese Longitudinal Healthy Longevity Survey (CLHLS)	7375	85.6	3316	83.7	87.2	0	0	7375	0	0	0	0
		65-113		65-111	65-113							
Detroit Neighborhood Health Study	845	59.3	333	57.8	59.9	685	97	3	7	0	19	26
		19.7-98.2		19.7-94.7	21.7-98.2							
Duke University Imaging and Genetics	331	19.25	142	19.27	19.23	48	196	97	9	0	26	32
		12.26-22		12.26-22	12.9-22							
Marshfield Clinic Personalized Medicine Research Project (PMRP)	2271	72.7	910	73.7	72.2	0	2192	4	0	0	8	12
		54.1-101.7		54.6-96.1	54.1-101.7							
Pediatric Imaging Neurocognition and Genetics (PING)	398	12.92	204	13.58	12.00	47	266	102	23	34	127	110
		3-22.7		3-22.7	3.17-22.58							
UCLA Consortium for Neuropsychiatric Phenomics	308	28.0	132	29	27	3	254	2	46	0	2	99
		21-50		21-50	21-50							

## Discussion and conclusions

There are a number of consortium efforts to standardize phenotypic measures to facilitate large-scale data sharing and comparison for genomic studies. The eMERGE network has shown that electronic algorithms can be developed and applied to electronic medical records to produce valid phenotypes for use in genome-wide association studies [12]. Similarly, the Phenotype Standardization Project is developing valid phenotypes for pharmacogenetic studies of serious adverse drug reactions [17-19]. The goal of the PhenX RISING network was to evaluate implementation of PhenX measures into ongoing genomic studies. We have shown the PhenX measures to be useful for large-scale studies linking genotypes and phenotypes and we identified a number of issues in the use of the PhenX Toolkit that were addressed to improve the Toolkit for future users. Advantages include the large number of measures employed and the diversity of administration and study cohorts. The diversity could also be viewed as a disadvantage because there was little replication for specific measures and study cohort types. Ongoing validation efforts at many of the sites will provide information about the accuracy of the data collected in various formats and with any modifications implemented at the sites.

Several cross-network analyses are ongoing between the groups that have collected the same PhenX measures. The Data Use Agreement and the standardized PhenX measures will facilitate these collaborations. The within- and between-group gene/environment analyses will be the ultimate test of the PhenX measures. Other researchers who use the PhenX measures are encouraged to provide feedback to RTI for continual improvement of the Toolkit.

## Competing interests

The authors declare that they have no competing interests.

## Authors’ contributions

CAM secured funding, oversaw data collection at Marshfield and drafted the manuscript. WH assisted with the data harmonization and table development. AEA secured funding, oversaw data collection at Michigan and assisted with manuscript preparation. RMB secured funding, oversaw data collection at Los Angeles, and assisted with manuscript preparation. AH secured funding, oversaw data collection at Duke and assisted with manuscript preparation. TLJ secured funding, oversaw data collection at UC San Diego and assisted with manuscript preparation. EN assisted with data collection at UC San Diego and assisted with manuscript preparation. DKS secured funding, oversaw data collection at Oklahoma, oversaw data collection and assisted with manuscript preparation. TJS secured funding, oversaw data collection at Duke and assisted with manuscript preparation. YZ secured funding, oversaw data collection in China and assisted with manuscript preparation. EMR managed the PhenX RISING network and assisted with manuscript preparation. HAJ assisted with management of the PhenX RISING network, data harmonization and manuscript development. All authors read and approved the final manuscript.

## Pre-publication history

The pre-publication history for this paper can be accessed here:

http://www.biomedcentral.com/1755-8794/7/16/prepub

## The PhenX RISING network members include

AIDHS/SDS site: Dharambir Sanghera, Ph.D., PI, Associate Professor of Pediatrics and Pharmaceutical Sciences, University of Oklahoma Health Sciences Center, Latonya Been, Research Coordinator, and Timothy Braun, Graduate Research Assistant and PhD candidate, Laboratory of Molecular Genetic Epidemiology, University of Oklahoma Health Sciences Center.

DNHS site: Allison E. Aiello, PhD, MS, University of Michigan School of Public Health, DNHS PI; Monica Uddin, PhD, Wayne State University, Co-Investigator; Helen C. S. Meier, MPH, DNHS Coordinator; Caroline Cheng, MS DNHS Biostatistician.

Duke University, Imaging Genetics site: Timothy Strauman, Ph.D., PI, Professor of Psychology and Neuroscience, Duke Institute for Brain Sciences; Ahmad Hariri, Ph.D., Co-PI, Professor of Psychology and Neuroscience, Investigator in the Institute for Genome Sciences & Policy, Director of the Laboratory of NeuroGenetics; Jared Minkel, Ph.D., Postdoctoral Research Fellow, Laboratory of NeuroGenetics.

Marshfield Clinic Personalized Medicine Research Project site: Catherine A. McCarty, PhD, MPH, Essentia Institute of Rural Health, overall PI; Murray Brilliant, PhD, Marshfield Clinic site PI; Marylyn D. Ritchie, PhD, Pennsylvania State University site PI; Terrie Kitchner, Marshfield Clinic Senior Research Coordinator; Carol Waudby, Marshfield Clinic Research Coordinator; Richard Berg, Marshfield Clinic Biostatistician.

## Pediatric Imaging, Neurocognition, and Genetics (PING) site

Part A: Infrastructure.

## Coordinating Core

Terry L. Jernigan, Ph.D., UC San Diego, Co-PI of PING, Core PI; Connor McCabe, B.S., UC San Diego; Erik Newman, Ph.D., UC San Diego.

## Assessment Core

Linda Chang, M.D., U Hawaii, Co-PI of PING, Core PI; Natacha Akshoomoff, Ph.D., UC San Diego.

## MRI Post-processing Core

Anders M. Dale, Ph.D., UC San Diego, Co-PI of PING, Core PI.

## MRI Acquisition Core

Thomas Ernst, Ph.D., U Hawaii, Co-PI of PING, Core Co-PI, Anders M. Dale, Ph.D., UC San Diego, Core Co-PI; Joshua Kuperman, Ph.D., UC San Diego.

## Genetics Core

Sarah Murray, Ph.D., Scripps Translational Science Institute, Co-PI of PING, Core PI; Cinnamon Bloss, Ph.D., Scripps Translational Science Institute; Nicholas J. Schork, Ph.D., Scripps Translational Science Institute.

## Informatics and Biostatistics

Mark Appelbaum, Ph.D., UC San Diego; Anthony Gamst, Ph.D., UC San Diego; Clint Cushman, UC San Diego; Tyler Smith, UC San Diego; Victor Rodgriquez, UC San Diego.

Part B: Investigators by PhenX Data Collection Site.

## University of California, San Diego

Terry L. Jernigan, Ph.D.; Anders M. Dale, Ph.D.; Natacha Akshoomoff, Ph.D.

## University of Hawaii

Linda Chang, M.D.; Thomas Ernst, Ph.D.

## University of California, Los Angeles

Elizabeth Sowell, Ph.D.

## Sackler Institute, Weill Cornell Medical College

B.J. Casey, Ph.D.; Erika J. Ruberry, B.A;. Alisa Powers, B.A.

## University of Massachusetts

Jean Frazier, M.D.; David Kennedy, Ph.D.

## Yale University

Jeffrey Gruen, M.D.

UCLA Consortium for Neuropsychiatric Phenomics.

## Consortium for Neuropsychiatric Phenomics, Coordinating Center (UL1DE019580)

Robert M. Bilder, PhD (Director/PI); Nelson Freimer, M.D. (Co-Director, Co-PI); Fred w. Sabb, Ph.D. (Scientific Project Manager)..

## Whole Genome Association Analysis Strategies for Multiple Phenotypes (RL1MH083268)

Nelson Freimer, MD (PI).

## Memory Mechanisms and Mental Disorders (RL1MH083269)

Tyrone Cannon, Ph.D. (PI).

## Response Inhibition and Dopamine Neurotransmission (RL1DA024853)

Edythe London, Ph.D. (PI).

## Translational Models of Memory and Cognitive Control (RL1MH083270)

J. David Jentsch, Ph.D. (PI).

## Hypothesis Web Development for Neuropsychiatric Phenomics (RL1LM009833)

D. Stott Parker, Ph.D. (PI).

## Human Translational Applications Core PL1MH083271

Robert M. Bilder, Ph.D. (Director/PI, and Director of Human Phenotyping Unit); Russell Poldrack, Ph.D. (Director of the Neuroimaging Unit); and Catherine Sugar, Ph.D. (Director of the Biostatistics and Database Management Unit).

## Translational Methods/Facilities Core (PL1NS062410)

Christopher Evans, Ph.D. (Director, PI); Nelson Freimer (Director, Genetic Studies Unit); J. David Jentsch (Director of Rodent Phenotyping Unit); X. William Yang (Director, Transgenic Models Unit).

## Chinese Longitudinal Healthy Longevity Survey (CLHLS)

Yi Zeng, Ph.D, Professor at Duke University Medical School and a Professor at Peking University (PKU). He divides his time between Duke and PKU, which makes CLHLS project co-funded by NIH and Chinese resources since 1998 much more feasible (e.g., he is fully eligible to apply for grants from both U.S. and Chinese resources as PI).

J. Vaupel, Ph.D, senior adviser, Professor at Duke University and Director of Max Planck Institute for Demographic Research.

L. George, Ph.D, senior investigator, Professor at Department of Sociology and Center for Study of Aging and Human Development, Duke University.

Huashuai Chen, Research Associate and CLHLS Database Manager, Center for Study of Aging and Human Development, Duke University.

Yuzhi, Liu, Scientific Manager of CLHLS field work in China (with a sub-contract to PKU), Senior Research Fellow and Deputy Director, Center for Healthy Aging and Development Studies, National School of Development, PKU.
